# Large Volume Osteosarcomas of the Femur Treated with Total Femoral Replacement

**DOI:** 10.5704/MOJ.1403.015

**Published:** 2014-03

**Authors:** KL Pan, WH Chan, P Shanmugam, GB Ong, F Kamaruddin, S Tan

**Affiliations:** Department of Orthopaedics, Faculty of Medicine and Health Sciences, Universiti Malaysia Sarawak, Kuching, Malaysia; Department of Orthopaedics, Faculty of Medicine and Health Sciences, Universiti Malaysia Sarawak, Kuching, Malaysia; Department of Orthopaedics, Faculty of Medicine and Health Sciences, Universiti Malaysia Sarawak, Kuching, Malaysia; Department of Paediatric Oncology, Sarawak General Hospital, Kuching, Malaysia; Department of Orthopaedics, Sarawak General Hospital, Kuching, Malaysia; Department of Radiology, Sarawak General Hospital, Kuching, Malaysia

## Abstract

Patients with extensive malignancies involving the femur often require total femoral replacement when their limbs can be salvaged. Reported series are small and involve heterogeneity of tumours. We present nine patients with osteosarcomas of the femur treated at our institution between 2003 and 2010 with a mean follow-up of 27 (6 to 56) months. Their ages ranged from 9 to 17 (mean 14 years). They had large volume tumours (mean 911 cm3) and presented late with a mean of 5.5 months from the onset of symptoms to definitive treatment. All patients underwent resection and total femur replacement. Six patients have died and two are alive with good function at the time of this report. One was lost to follow-up. These patients require a high level of treatment care and have a guarded prognosis.

**Key Words:**

Large osteosarcomas, femur, total femoral replacement

## Introduction

Total femur replacement is not commonly done. There are a few series in the literature and their numbers are small. When indicated for malignancies involving the femur, it usually means that the tumours are extensive. In the East Malaysian states of Sarawak and Sabah, many patients still present late when their tumours are large “football-sized” lesions. Many come from the rural, inland highlands where medical services are scarce and not easily accessible. The majority seek treatment initially from village traditional “medicine-men”. Only when the tumours are huge or the pain unbearable do they seek hospital care. We present nine such patients requiring total femur replacement and their outcome.

## Materials and Methods

### 

Between 2003 and 2010, nine patients with large osteosarcomas of the femur treated at our institution were retrospectively reviewed. Each of them underwent resection of the tumour together with removal of the whole femur and reconstruction with a total femoral endoprosthesis. There were six males and three females aged between nine and 17 years (average 14 years). Their progress was charted for an average of 27 months (range, 6 to 56); until death or the last follow-up. One patient was lost to follow-up and not contactable at 10 months.

Staging was done with plain x-rays of the femur, chest x-ray, MRI of the lesion, CT thorax and bone scan. The MRI included the whole femur to look for skip lesions and measurements of the length, width and depth of the tumour; from which the volume was calculated. This was based on the mathematical formula for an ellipsoid: V = 4/3 pi (L/2 x W/2 x H/2) where W = Width, H = Height, L = Length, and V = Volume1,2. In one patient, the parameters were obtained from the excised tumour specimen. The pre-operative diagnosis was established with an incisional biopsy.

Two or three cycles of neoadjuvant chemotherapy were started, followed by a repeat MRI and-, definitive surgery, and another three or four cycles of adjuvant chemotherapy. Resection of the tumour and femur was done through an anteromedial curvilinear incision from the greater trochanter laterally to the medial aspect of the tibial tuberosity, including the biopsy scar in the process. The femoral vessels and sciatic nerve were isolated and the tumour and femur excised with an oncological margin. Reconstruction was done with modular tumour prostheses in eight patients (United Orthopaedic Corporation Tumour Implant) and a custom- made prosthesis in one. The prostheses had bipolar hips and rotating hinged knee joints and two were expandable (Eagle Osteon Expandable Tumour Prosthesis).

At the hip joint, the bipolar cup was contained in the joint capsule which was sutured with care to prevent postoperative dislocation. The hip abductors, with or without a sliver of bone, were sutured with non-absorbable sutures to the prosthesis. The patella and extensor mechanism of all patients were preserved. The vastus intermedius was always resected together with the tumour as well as part of the vastus lateralis or medialis, depending on the location of the tumour. The rectus femoris was not involved in the tumour in all nine patients, allowing preservation of the extensor mechanism. Subsequent rehabilitation took a longer time when more muscle bulk was removed. The whole resected specimen was submitted for histopathological examination to reconfirm the histological type, verify margins and determine the percentage of tumour necrosis (Table I). Postoperatively, the patient was nursed with hips abducted and the knee splinted in extension. Static quadriceps exercises were started as soon as possible. At two weeks on, the patient was allowed to ambulate with crutches when active straight leg raising was achieved. Knee flexion was then allowed gradually. After discharge, the patients were followed up regularly (three monthly) in the clinic with thigh and chest x-rays, and six-monthly CT thorax.

## Results

Eight patients had tumours arising from the distal femur (Fig 1) and one from the proximal third. The main symptoms were pain and swelling, with pain presenting first in eight patients. The average duration from the first noticed symptom to the time of definitive treatment was 5.5 months (range one to 14 months). Six patients visited traditional “healers” where their painful areas or swellings were massaged.

The average volume was 911 cm3 (range, 440 to 1527). The dimensions ranged from 15x8x7cm to 18x13.5x12cm (Table I). Three patients had skip lesions proximal to the tumour. The average distance of the proximal intramedullary extent or skip lesion to the greater trochanter was 5.4cm. Five patients were in the growing age of 9-14 years.

Two patients already had lung metastases at diagnosis and one had bone metastasis. According to the Musculoskeletal Tumor Society Staging System, six patients were in stage IIB and three stage IIIB. The average time taken for each surgery (not including anaesthetic induction and reversal time) was nine hours and 44 minutes (range- 7 hours 5 minutes to 13 hours). From the histopathological examination, six had osteoblastic osteosarcoma, two chondroblastic and one fibroblastic. In one patient, the margin was not clear (histopathological examination). Four patients developed new lung metastases after treatment, besides the two who already had lung lesions at time of diagnosis. Two patients had new bone metastases. There was one local recurrence (same patient whose margin was not clear). One patient had deep infection which cleared with antibiotics and two debridements. There has been no recurrence of the infection after three years but the knee was stiff. One patient was lost to follow-up at 10 months. One patient died from blood sepsis two weeks after the last chemotherapy cycle. Of the remaining seven patients, five have succumbed to the disease (mainly due to lung metastasis). The average survival time of this group was 25 months (range 7 to 56). Two patients were alive and well, at 43 and 56 months, with no evidence of disease at the time of this report. Their MSTS functional score was 28 (93%) and 27 (90%). All the patients had a Trendelenburg gait when they started to ambulate; which partially improved with time. Four patients had full active extension of the knee. Four had extensor lag of 5 to 10 degrees. Five patients were able to flex the knee more than 70 degrees. Two had flexions of 45 and 50 degrees and two were able to flex only 10 degrees (one of them had deep infection). There was no hip dislocation.

## Discussion

Patients with large volume osteosarcomas involving the femur which require total femur replacement are not common, with few reported series3. These reports included different types of malignant bone tumours and some with soft tissue tumours3,4,5,6,7. All our patients had primary, high grade osteosarcomas involving the femur. There is probably no consensus on what constitutes a large bone sarcoma in the femur. The TNM classification of bone tumours has 8cm as the demarcation between “small” and “large” tumours. All our patients had tumours much larger than this (Table I). The average volume calculated was 911 cm3 (range, 440 to 1527), bearing in mind that five of them were still in the growing age group (height and limb length less than adults). The proximity of the intramedullary extent or skip lesion to the greater trochanter (average 5.4cm) was the critical factor for the - requirement of a total femur replacement; when a distal femoral prosthesis was not feasible due to the short arm of the proximal segment. The main reason for the extensive tumour at presentation was probably the long delay (average 5.5 months) between onset of the first symptom to definitive treatment. The majority (6) also had traditional treatment involving massage to the swelling. This would have brought in more blood supply and stimulated the growth of the tumour, besides increasing the risk of metastasis. Many lived in remote areas where the first visit for an ailment was to a village “healer”. When there was no improvement, the patients would visit a government health clinic usually manned by medical assistants or nurses. After a few visits, they would be sent to a district hospital and seen by a junior medical officer. When painkillers and sometimes antibiotics do not work, an x-ray would finally be taken and a provisional diagnosis of a sinister bone lesion made; at which point, they will be sent to a larger regional hospital for an MRI before being sent to a tertiary centre. Each step required expenses, mainly for transport and the accompanying person, contributing to the delay.

We were apprehensive of postoperative infection from the long surgery (average 9hr 40 min), large incision with skin flaps that had to be raised, and a large metal implant; in addition to the ongoing chemotherapy. However, there was only one female patient with infection, which cleared after debridement and antibiotics. After 43 months, there was no further evidence of infection but range of motion of her knee is markedly limited from the resulting fibrosis. The femoral/popliteal vessels were tightly stretched by the tumours with multiple small vessels branching off into the tumours. Securing these small vessels and releasing the main vascular bundle contributed to the prolonged surgery. The vessels also made “indentations” into the tumours and at these places, the gross margins were tenuous although the only local recurrence was in the patient in whom we had breached the margins of the tumour and contaminated the operative field.

Three patients already had distant metastases at presentation, two to the lungs and one to the bone. We proceeded with full treatment after consultation with the parents and on the premise that osteosarcomas responded to chemotherapy. One patient with lung secondaries died from generalized sepsis, probably from the complications of chemotherapy (two weeks after the last cycle). His blood culture was positive and there was no evidence of local infection at the operative site. Another patient, a female, with lung metastases had operative resection of the nodules. She was subsequently treated with a second and third line of chemotherapy, but died of the disease at 33 months. The patient with bone secondaries at T7 had a corpectomy. He died of lung lesions at 11 months. Four patients whose CT thorax was clear at presentation developed lung metastases during follow-up. Another two developed bone lesions. One patient had local recurrence. He died from lung metastasis at seven months. Of the nine patients, one was lost to follow-up, six had died and only two are alive and free of disease (2/9) at 43 and 56 months. This compares with other reports of the survival of osteosarcoma patients with total femoral replacement within their series: Neurabay8 3/13; Ward9 3/7; Ruggieri6 6/11. Both the- surviving patients in our series are ambulating well without pain and have MSTS scores10 of 90% and 93%.

**Table I: Data of Patients with Total Femoral Replacement:**
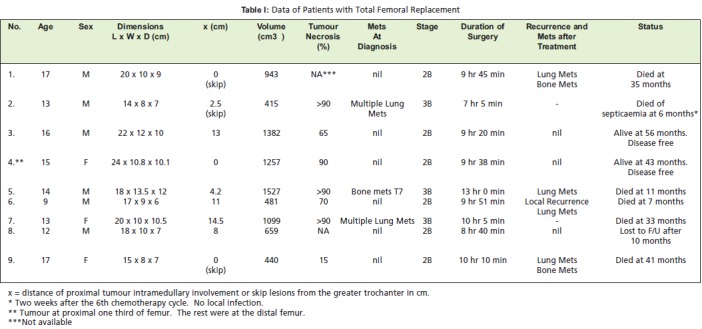


**Figure I: Compilations of Images:**
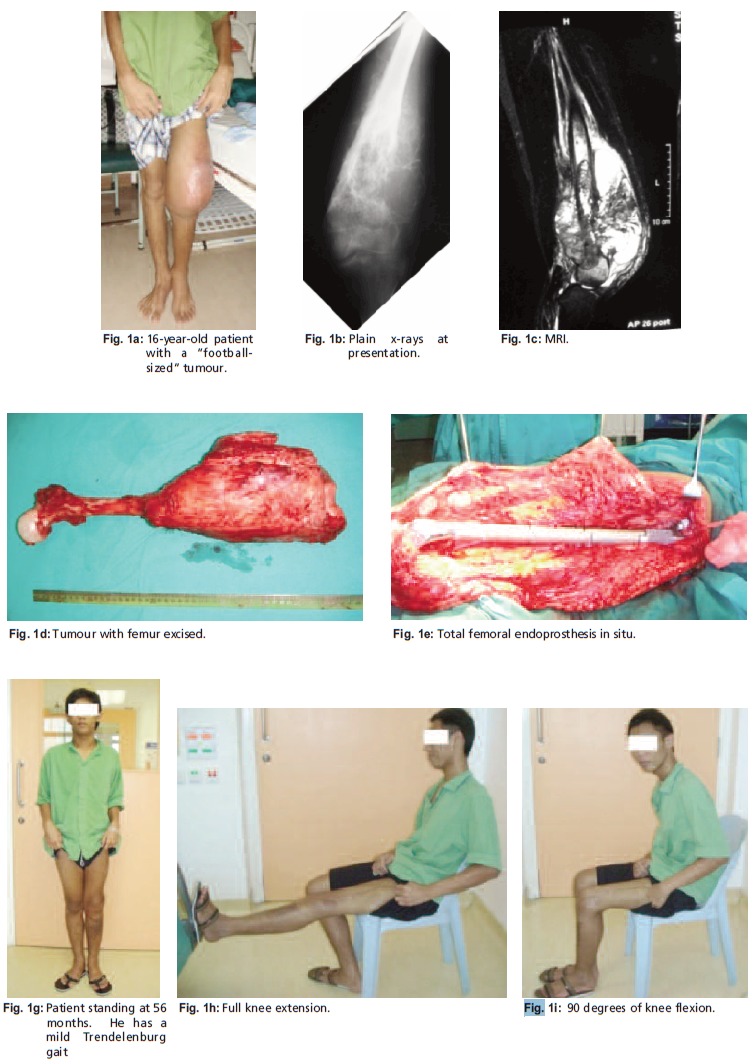


## Conclusion

The small number of patients does not allow us to make firm conclusions. Patients with large volume osteosarcoma of the femur with total femoral replacement require a high level of concentrated care from many disciplines and at great expense. They can achieve good function if they survive. Perhaps more effort should be made at reducing delays in presentation at tertiary care centres so that they do not require a total femur replacement in the first place.

## References

[1] Schmidt KF, Ziu M, Schmidt NO, Vaghasia P, Cargioli TG (2004). Volume reconstruction techniques improve the correlation
between histological and in vivo tumor volume measurements in mouse models of human gliomas. J Neurooncol.

[2] Kim MS, Lee SY, Cho WH, Song WS, Koh JS, Lee JA (2009). Effect of increases in tumour volume after neoadjuvant
chemotherapy on the outcome of stage II osteosarcoma regardless of histological response.. J Orthop Sci.

[3] Ahmed AR (2010). Total femur replacement. Arch Orthop Trauma Surg.

[4] Kaira S, Abudu A, Murata H, Grimer RJ (2010). Total femur replacement: Primary procedure for treatment of malignant tumours
of the femur. Eur J Surg Col.

[5] Natarajan MV, Balasubramaniam N, Jayasankar V, Sameer M (2009). Endoprosthetic reconstruction using total femoral custom
megaprosthesis in malignant bone tumours.. Int Orthop.

[6] Ruggieri P, Bosco G, Errani C, Mercuri M (2010). Local recurrence, survival and function after total femur resection and megaprosthetic
reconstruction for bone sarcomas.. Clin Orthop Relat Res.

[7] Yoshida Y, Kojima T, Taniguchi M, Tokuhashi Y (2012). Experiences with total femur replacement for malignant bone and soft
tissue tumors. Asian Biomed.

[8] Nerubay J, Katznelson A, Tichler T, Rubinstein Z, Morag B, Bubis JJ (1988). Total femoral replacement.. Clin Orthop Relat Res.

[9] Ward WG, Dorey F, Eckardt JJ (1995). Total femoral endoprosthetic reconstruction. Clin Orthop Relat Res.

[10] Enneking WF (1983). Staging musculoskeletal tumors. In: Enneking WF (ed). Musculoskeletal tumor surgery. Qual Life Res.

